# Who benefits most from influenza vaccination policy: a study among the elderly in Beijing, China

**DOI:** 10.1186/s12939-016-0332-x

**Published:** 2016-03-08

**Authors:** Tongtong Li, Min Lv, Trudy Lei, Jiang Wu, Xinghuo Pang, Ying Deng, Zheng Xie

**Affiliations:** School of Public Health, Peking University, Beijing, 100191 P.R. China; Beijing Center for Disease Prevention and Control, Beijing, 100013 P.R. China; Columbia University Mailman School of Public Health, New York, 10032 USA

**Keywords:** Rural and urban disparity, The elderly, Concentration index, Developing country

## Abstract

**Background:**

Influenza continues to have a major impact on vulnerable populations worldwide, particularly among the elderly (≥60 years of age). Vaccination for targeted groups is recommended by the WHO as the most effective way to control influenza infections. Since 2009, the Beijing municipal government has provided influenza vaccination to the elderly at no out-of-pocket cost to reduce influenza threats and improve related health equality. The study aims to evaluate the equality of the policy, and to analyze factors that bring influences to equality.

**Methods:**

Based on data from a household survey, concentration index (CI) was calculated to measure the socioeconomic inequality in influenza vaccination. A Logit regression model was used to decompose CI, in which the contribution of each determinant was calculated and the percentages of these contribution were obtained.

**Results:**

Free influenza vaccination at point of use shows significant pro-poor distribution among the elderly in Beijing (CI = −0.115). After the decomposition of CI, the elderly with lower income, higher education, and living in rural areas were more likely to get the influenza vaccination, in which place of residence (contribution percentage = 57 %) held the most contribution of variance.

**Conclusions:**

Beijing’s free influenza vaccination strategy at point of use could provide the poor elderly with equal opportunities to receive preventive health service, showing a significant pro-poor distribution. The poor elderly, who live in rural areas with high education, benefit most from the policy. Further policy interventions should target the population living in urban areas in order to improve the utilization of public health services and health equality.

## Background

Influenza is a preventable, communicable viral illness [1], resulting in major social, personal, and economic burdens worldwide [2]. As much as 5–15 % of the population is affected by influenza annually [3], with an even higher rate during epidemics. Among these, the elderly (≥60 years old) appear to be at the highest risk of influenza infection and death— at least one in every 300 elderly adults is hospitalized due to influenza each year and they constitute 90 % of all influenza-related deaths [4]. Influenza vaccination is the most effective method of reducing the morbidity and complications of influenza infections [5]. Thus, the World Health Organization (WHO) recommends annual vaccination against influenza for elderly individuals [6].

Immunization against influenza is considered to be the most important primary health intervention to control influenza epidemics. Many countries have tried to implement policies to increase the coverage and equality in the utilization of influenza vaccination successfully [7], but due to limited health finances, influenza vaccination often only targets high-risk individuals such as the elderly [8]. These target populations are encouraged with financial subsidies or other strategies to receive the influenza vaccine, such as in the United States [9], Canada [10], and Japan [11]. Among the related polices, providing the vaccination at no out-of-pocket expense is always thought to have a strong positive impact on vaccination coverage rates [12] as well as on equality across the target population, playing an important role in influenza control overall.

In most developed countries, inequalities in health care distribution tend to have a pro-poor bias, meaning lower income groups use health care services more than the rich groups [13–15]. However, studies in some low- and middle-income countries (e.g., China) have found that there are pro-rich inequalities in most of the primary health service utilization [16, 17], due to differences among countries’ capacities, priorities, and resources to establish suitable policies and strategies and implement those policies. During the last decade, there has been growing interest in reducing health inequities globally [18], however, there are still wide disparities in influenza vaccination coverage in most developing countries [19, 20]. Comprehensive influenza vaccination provision is key to improve equality, or at least to construct a pro-poor distribution, and should be given more political and financial support.

In China, influenza vaccination is not prioritized in public health and not included in the immunization program in most localities. Therefore, it generally does not receive any governmental reimbursement. People must purchase influenza vaccines themselves, resulting in a barrier against influenza vaccination coverage and equality [21]. However, since 2009, the Beijing municipal government has provided free influenza vaccines at point of use (hereafter termed as free influenza vaccines), which is financed by municipal taxation to the elderly (≥60 years old) to reduce influenza threats and improve related health equality. As Beijing is the first city to carry out this policy in China, limited studies regarding it are available. Therefore, this study aims to describe the impact of the free influenza vaccines, determine whether such policy is progressive or regressive. We also aim to find out the relevant factors in achieving equality during the implementation of the policy.

## Methods

### Data

As prior studies showed [22, 23], the influenza vaccination coverage rates for the elderly (≥60 years old) in Beijing (*p*) is about 40 %, with α at 0.05 (two side test) and a permissible error (*δ*) of 0.05, yielding a sample size of 368 by (1):

1 $$ n={\left({\scriptscriptstyle \frac{\mu_{\alpha }}{\delta }}\right)}^2p\left(1-p\right) $$

Considering a dropout rate of 50 % in total and maybe an even lower response rate in urban areas, a sample size of 1472 questionnaires was calculated to obtain accurate estimates for influenza vaccination coverage rates.

This study used a multistage, stratified, random, sampling design. Since 2005, Beijing’s 16 districts have been divided into 4 belts by the local government, according to geographic location, economic development status and function in the city [24]. Within each belt, 2 districts were selected randomly through consideration of their representativeness and sample size, totaling 8 districts. Then, probability proportionate to size sampling (PPS) method [25] was used to select 8 communities in each district, considering the population of the elderly. A name list of all the elderly who had lived at each community for more than half years, was provided by local administration. The participants (≥60 years old) were selected by systematic sampling according to this list, with the initial subject determined by a random number. Though the total population of the elderly in each community was varied, 30 participants in each urban community and 25 participants in each rural community were sampled. Participants who had severe psychosis diseases or were not willing to take part in this survey were excluded. The final sample consisted of 1685 eligible participants and 1628 were enrolled in the analysis of this paper eventually with a response rate of 96.6 %.

### Ethics approval

Ethics approval for this study was obtained from Peking University Health Science Center in China (protocol number IRB00001052-13080).

### Measures

This study was conducted in June 2013. A questionnaire with 56 questions was designed by the research team. The questionnaire took around 20 min for respondents to complete. Within the questions, influenza vaccine acceptance was assessed by: “Did you accept the free influenza vaccine last year?” Per capita household income was used as a proxy for expected socioeconomic level, which assessed by “How much money did your household earn monthly on average?” and “How many family members in your household?” Place of residence was measured by one question: “Where do you live?” with response categories ‘urban’ and ‘rural’. Furthermore, age, gender, and education level were included as control variables.

### Analyses

To measure horizontal inequality and explain socioeconomic-related inequality in the utilization of free influenza vaccine, calculation of concentration index (CI), a method proposed by Wagstaff and van Doorslaer, was used [26, 27]. Using the concentration curve, which plotted the cumulative percentage of the utilization of the free influenza vaccine on the y-axis against the cumulative percentage of the population ranked by per person monthly income from poorest to richest on the x axis, the concentration index value was calculated [28]. More concisely, the concentration curve plotted segments of the health variable against quantiles of the living standards variable. Then the concentration index was defined as two times the area between the concentration curve and the line of equality (the 45° line) [29]. As per convention, the CI takes a negative value when the curve rises above the line of equality, indicating disproportionate concentration of the health variable among the poor compared to rich, and a positive value when it falls below.

For ease of explanation, the concentration index was decomposed into individual factors contributing to income-related health inequality, in which each contribution factor is the degree of income-related inequality. The decomposition of concentration index was given by (2):

2 $$ \mathrm{C}\mathrm{I}={\displaystyle {\sum}_k{\scriptscriptstyle \frac{\beta_k{\overline{X}}_k}{\mu }}}C{I}_k+{\scriptscriptstyle \frac{GC{I}_{\varepsilon }}{\mu }} $$

where all the cases in the sample were assumed to share the same coefficient vector, β_κ_. ${\overline{X}}_k$ is the mean of X_κ_, CI_κ_ is the concentration index for X_κ_, and GCI_ε_ is the generalized concentration index for the error term. The Logit regression model was used to decompose the concentration index, in which the contribution of each determinant to the CI was calculated and the percentages of these contribution were obtained. All analysis was performed using Stata12.0.

## Results

### Descriptive statistics of the sample

Table 1 shows the socioeconomic characteristics of the elderly in this household survey. On average, the population were 70.0 (±7.062) years old and earned 2203.0 (±1394.491) CNY monthly per person (Table 1). The sex ratio of our participants slightly skewed towards female (56.8 %). It is notable that majority of the participants lived in urban areas (83.5 %).

**Table 1:** Descriptive statistics

Variables	Percentage (%)	S.D.
Age (years)	70.0	7.062
Gender		
Male	43.2	0.012
Female	56.8	0.012
Place of residence		
Urban	83.5	0.009
Rural	16.5	0.009
Per capita household income(CNY)	2203.0	1394.491
Education		
≤ Primary school	32.8	0.012
Junior high school	30.3	0.011
Secondary school	20.3	0.010
Beyond secondary school	16.6	0.009

### Concentration index of acceptance

**Fig. 1:**
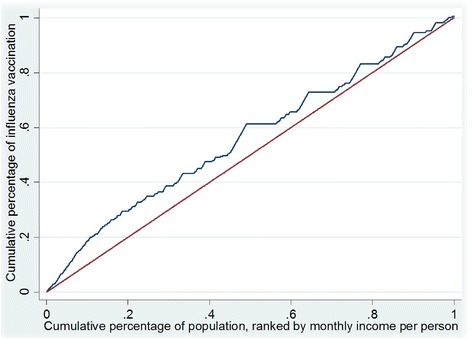
Concentration curve of influenza vaccination among the elderly in Beijing, China

There was an obvious negative relationship between per capita household income and influenza vaccination coverage (%): the higher the income quintile, the lower the vaccination coverage. This trend was further confirmed by the concentration curve of vaccination coverage, which laid above the line of equality (Fig. 1), and the CI value was rounded to −0.115. These results demonstrate that the influenza vaccination was unequally distributed among the Chinese elderly population and was more prevalent among those with lower income.

### Decomposition of concentration index

#### Concentration index of determinants

Table 2 shows the concentration index values of determinants, whose calculation and explanation is consistent with the concentration index of influenza vaccination. Among these determinants, participants who were males, lived in urban areas, and held a secondary school degree or higher had a higher likelihood of having more financial resources. Older persons tended to be slightly richer than their younger counterparts. The concentration index per capita household income has a similar meaning to the Gini coefficient [30] and has a value of 0.139 (95 % confidence interval: 0.126, 0.152) indicating that it is relatively fair for the overall income inequalities in Beijing’s influenza policy for the elderly.

**Table 2:** Concentration index of determinants

Variables	CI_k_	95 % CI	
Age (years)	0.008	0.005	0.010
Gender	−0.013	−0.022	−0.005
Place of residence	−0.0003	−0.010	0.009
Per capita household income (CNY)	0.139	0.126	0.152
Education			
≤ Primary school	−0.059	−0.100	−0.018
Junior high school	−0.122	−0.164	−0.081
Secondary school	0.069	0.016	0.123
Beyond secondary school	0.256	0.197	0.314

#### Regression analysis

Table 3 shows the results of logit regression model on influenza vaccination among the elderly in Beijing. Among the influencing variables, age and per capita household income were continuous variables, with older (0.031) and poorer (-0.000108) groups being more likely to accept the free influenza vaccines. As for categorical variables, living in rural areas (1.214) and better education increased the probability of receiving the free influenza vaccine.

**Table 3:** Logit regression results of factors influencing influenza vaccination

Variables	β_k_	95 % CI	
Age (years)	0.031**	0.015	0.047
Gender			
Male (ref)			
Female	−0.212	−0.425	0.001
Place of residence			
Urban (ref)			
Rural	1.214**	0.900	1.528
Per capita household income(CNY)	−0.000108*	−0.000201	−0.000015
Education			
Beyond secondary school (ref)			
≤ Primary school	−0.396*	−0.761	−0.030
Junior high school	−0.634**	−0.976	−0.292
Secondary school	−0.401*	−0.746	−0.056

*P < 0.050, **P < 0.001

#### Contributions of determinants

**Fig. 2:**
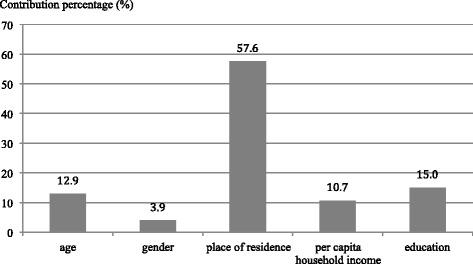
Contribution percentages of determinants to concentration index of influenza vaccination among the elderly in Beijing, China

The contribution of each determinant to the concentration index of influenza vaccination among the elderly was calculated and the percentages of these contributions were further obtained by dividing the contributions by concentration index of influenza vaccination. The contribution percentages are plotted in Fig. 2. Place of residence had a dominant contribution (57.6 %), followed by education (15.0 %), age (12.9 %), per capita household income (10.7 %), and gender (3.9 %).

## Discussion

In this paper, individual factors affecting the acceptance and uptake of influenza vaccination after the implementation of the policy for free influenza vaccines for the elderly in Beijing, China have been explored. Our findings suggest that free access to influenza vaccination for the elderly in Beijing has a negative correlation with economic status, showing a pro-poor distribution. This confirms that the policy has the ability to target the poor in providing benefits, though not all health care policies are able to achieve this positive impact [31–34]. The pro-poor distribution under such policy may be attributed in part to two vital factors: governmental willingness and the implementation of the policy and its services. As it is quite clear that policies that eliminate out-of-pocket expenses can improve health equality, several governments have therefore designed such policies [35–37]. Meanwhile, suitable implementation is also essential to reach the target populations and make sure the health care services are accessible, particularly to poorer populations [38]. As the Beijing municipal government successfully passed an appropriate policy that provided adequate services to the vulnerable population, it is important to understand the relevant factors to focus on for implementation to enhance results.

Our results show that there is no correlation between gender and free influenza vaccination in Beijing, implying that the old women and men enjoy the same opportunities under the policy. Since achieving gender equality in health outcomes an important principle worldwide [39], this free influenza vaccination policy showed a positive influence on primary health care services delivery. Regarding age however, although influenza vaccination is targeted toward all the elderly (≥60 years old) as per the rules of the Beijing Health Bureau, those at the younger spectrum of this age group are less likely to receive the vaccination in this study, in line with results of previous studies [40]. This suggests that more strategies should be implemented to ensure that vaccines are reaching the entire target population.

The findings illustrate that place of residence of the elderly in Beijing has the strongest correlation with the uptake of free influenza vaccines, with individuals who live in rural areas more likely to receive this health service. This is in line with the previous study on the same policy [41], but quite different from other studies conducted in other cities in China [42]. The differing result may be due to the more effective mobilization and the closer doctor-patient relationship in rural areas in Beijing. Though the influenza vaccination policy has the same user fee reimbursement in both targeted rural and urban areas, influenza vaccine uptake mobilization differs according to geographic community characteristics [21]. Most rural community members have lived near to each other for a long time and communicate more than those who live in urban areas [43]. As such, every eligible individual in rural areas likely receives influenza vaccination information more directly and easily. On the other hand, the organization of vaccination in urban communities is more difficult and less efficient, posing as a barrier to influenza vaccine uptake. Similarly, doctors are more familiar with local residents in rural areas due to the close-knit nature of the community [44, 45], resulting in stronger doctor-patient relationships than in urban areas [46, 47]. Thus, rural doctors can provide more health education and personal recommendations and patients are more respectful and trusting of the doctors’ recommendations, thereby promoting uptake of the free influenza vaccine.

The study shows that elimination of a user fee is beneficial to the financially disadvantaged elderly, increasing their utilization to influenza vaccination in Beijing. The results are significantly different from prior studies on other primary health care services in China, most of which have shown a pro-rich utilization [48–50]. This difference is likely because the elderly with higher incomes may be more concerned about the quality of health care services in comparison to the poorer elderly. Researches have shown that a fear of side effects and doubts about efficacy are of great importance to influenza vaccination acceptance [51]. Additionally, fee charging is also often associated with the quality of health care services [52]. Free services unintentionally signals low quality to individuals with higher income. Under such circumstances, even if richer elderly individuals are willing to accept influenza vaccination, they are more likely to pay for those of their own choosing [53], rather than accept those that are free-at-point-of-use. Thus, acceptance of health care services with no user fee, especially invasive therapy like vaccines, decreases among the elderly with higher income.

The results show that education level of the elderly is positively correlated with vaccination uptake at an even stronger degree than economic status, indicating the crucial nature of education level in health service disparities, though it is not in accord with the previous study on the same policy [23]. More education likely confers a better understanding of health risks and greater demand for suitable methods of prevention, leading to more effective utilization of health care services [54–57]. Furthermore, our results also show that the elderly with an education beyond secondary school are more likely to accept this free influenza vaccination, while other individuals with lower education are at almost the same level of vaccination acceptance, indicating university/college level education is essential for primary health care utilization. Interestingly, all the participants in this study were born before 1953, right at the early days of New China. During that time, the education system was very backwards, with a quite low rate of university/college enrollment at only 0.26 % [58]. During the 1970s, when the participants in this study should go to university/college, the rate of university/college enrollment increased only slightly to 1.6 %. However, along with the rapid social and economic development since the establishment of New China, the education system has undergone dramatic changes with the rate of university/college enrollment increasing to 37.5 % in 2014 [59]. As the proportion of the population with an education beyond secondary school increases in China, inequality in health services utilization caused by education level will be reduced in the future. Together, this change shows positive steps in education advancement towards the improvement of health care equality.

There are several limitations in this study. Firstly, the cross-sectional nature of the study dictates that only correlation, rather than causation, can be studied. Secondly, randomly selected non-respondents have not been included in the analysis. Most of them expressed their unwillingness to participate. The characteristics of non-respondents were not clear since we do not have sufficient information of them. This may cause selection bias [60]. Further study should explore the difference between respondents and non-respondents. Thirdly, we used stratified random sampling when sampling eight districts. However, this sampling method ignored the population size of the elderly in each district. If PPS were used at this stage, the sample procedure in this study would be improved. Finally, the conclusion drawn from this study may not apply to other targeted groups for influenza vaccination, such as children and pregnant women, due to the special socioeconomic characteristics of the elderly. In the future, it would be useful to perform more comprehensive studies to further evaluate the equality as well as other associated factors of the free influenza vaccination policy in Beijing to inform strategies for increasing primary health care equality.

## Conclusion

To conclude, Beijing’s free influenza vaccination strategy is of great importance for providing the poor elderly with equal opportunities to receive preventive health service. The policy shows significant pro-poor distribution among the elderly in Beijing, providing services targeted to those with lower income, higher education and live in rural areas. Such results are also relevant for future efforts of improving utilization to health services for the elderly in China. Further policy interventions should be aware of the weaknesses in provision of free public health services in urban settings and thus, target the urban population in order to improve the utilization of public health services.

## Abbreviations

CI: Concentration Index
CNY: Chinese Yuan
PPS: probability proportionate to size sampling
